# A Prokaryotic Twist on Argonaute Function

**DOI:** 10.3390/life5010538

**Published:** 2015-02-16

**Authors:** Sarah Willkomm, Adrian Zander, Alexander Gust, Dina Grohmann

**Affiliations:** 1 Institute of Molecular Medicine, Universitätsklinikum Schleswig-Holstein, University of Lübeck, 23538 Lübeck, Germany; E-Mail: willkomm@imm.uni-luebeck.de; 2 Physikalische und Theoretische Chemie-NanoBioSciences, Technische Universität Braunschweig, Hans-Sommer-Strasse 10, 38106 Braunschweig, Germany; E-Mails: a.zander@tu-bs.de (A.Z.); a.gust@tu-bs.de (A.G.)

**Keywords:** Argonaute, archaeal, interference, guide strand

## Abstract

Argonaute proteins can be found in all three domains of life. In eukaryotic organisms, Argonaute is, as the functional core of the RNA-silencing machinery, critically involved in the regulation of gene expression. Despite the mechanistic and structural similarities between archaeal, bacterial and eukaryotic Argonaute proteins, the biological function of bacterial and archaeal Argonautes has remained elusive. This review discusses new findings in the field that shed light on the structure and function of Argonaute. We especially focus on archaeal Argonautes when discussing the details of the structural and dynamic features in Argonaute that promote substrate recognition and cleavage, thereby revealing differences and similarities in Argonaute biology.

## 1. Introduction

**Figure 1:**
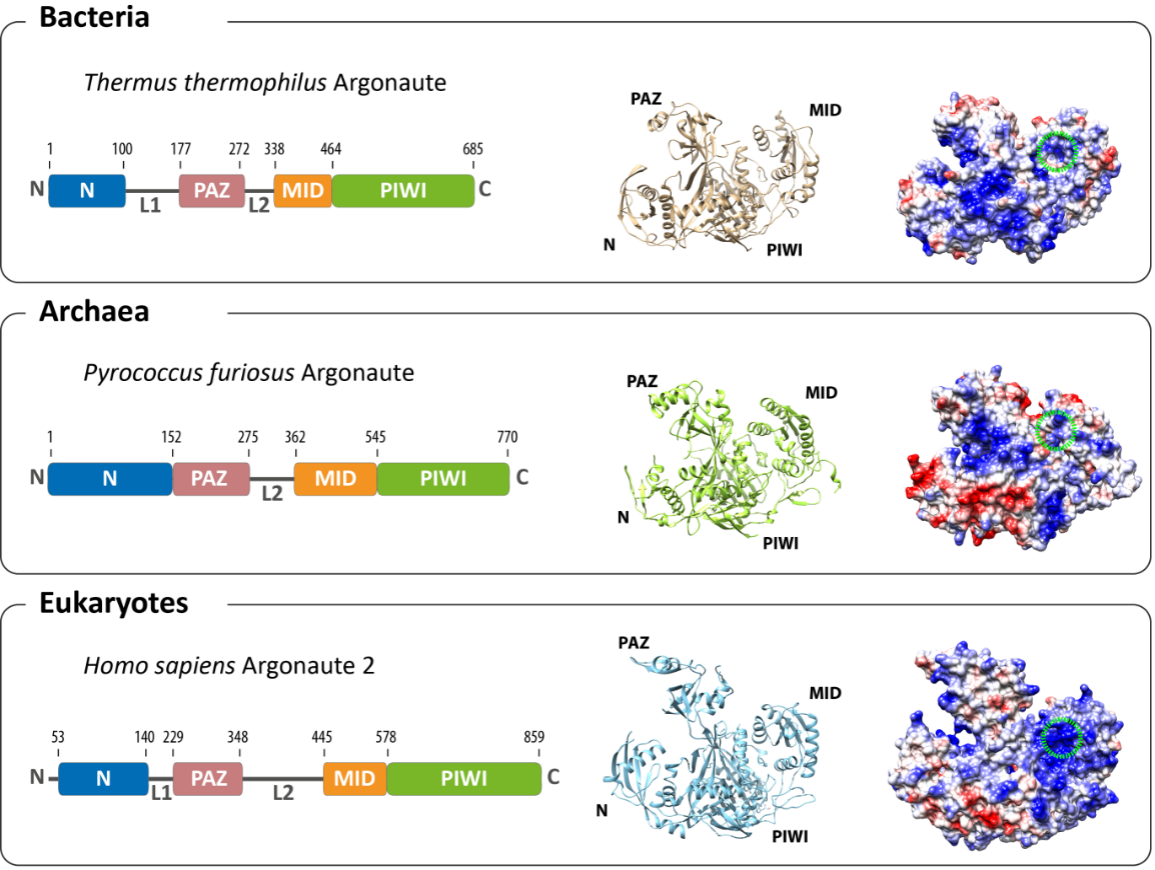
Overall architecture of Argonaute from the three domains of life. The domain composition (left) and structures (middle) of the bacterial (based on *Thermus thermophilus*, PDB: 3DLH), the archaeal (based on *Pyrococcus furiosus*, PDB: 1U04) and the eukaryotic (based on human Argonaute 2, PDB: 4EI3) Argonaute reveal an evolutionarily conserved architecture. Differences can be found in the surface charge distribution of Argonaute proteins (negatively-charged surfaces in red; positively-charged surfaces in blue). The binding pocket for the 5'-end of the guide in the MID domain is highlighted with a green circle.

**Figure 2:**
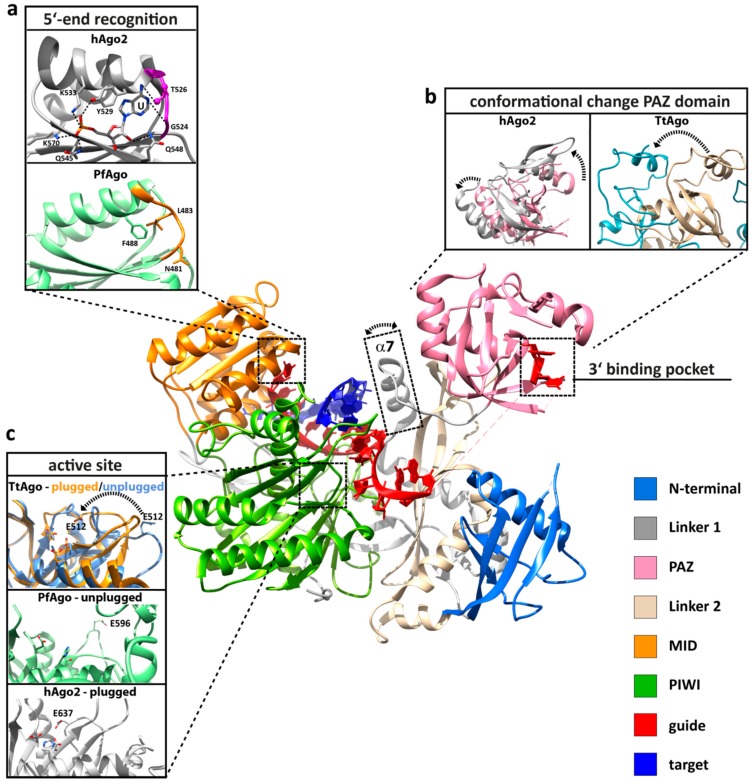
Important structural, functional and dynamic features of Argonaute. Structural elements that are important for Argonaute function are highlighted based on the human Argonaute 2 (hAgo2) structure in complex with a guide (red) and target (blue) strand (PDB: 4W5T). (**a**) The 5'-end is buried in a binding pocket in the MID domain (orange), where specific interactions with the terminal phosphate of the guide strand and interactions between the protein backbone of the specificity loop (highlighted in purple or orange) contribute to the specific recognition of the first nucleotide (PDB: 3LUD). This interaction network leads to the stable positioning of UTP in hAgo2. In contrast, the Argonaute structure from *Pyrococcus furiosus* (PfAgo) shows that the specificity loop (orange) is pulled away from the first nucleotide (PDB: 1U04). (**b**) The PAZ domain (pink) of all Argonaute variants is a mobile element, as revealed by structural, kinetic and single-molecule studies. Shown are the conformational changes (highlighted by a broken arrow) of the PAZ domain between the RNA guide-associated hAgo2 (pink, PDB: 4EI3) and hAgo2 in complex with an RNA guide and an 11-nucleotide RNA target (grey, PDB: 4W5T). The movement of the PAZ domain is more pronounced when comparing the structure of DNA guide-associated *Thermus thermophilus* Ago (TtAgo, PDB: 3DLH) and the ternary TtAgo complex, which also includes a 19-nucleotide RNA target (PDB: 3HVR). Progression to the ternary complex leads to the release of the 3'-end of the guide from its binding pocket in the PAZ domain. Another flexible element that undergoes a structural change upon ternary complex formation is helix α7 (boxed), which is only found in archaeal-eukaryotic Argonautes. (**c**) The PIWI domain (green) harbors the active site where the glutamate finger can be found in an “unplugged” or “plugged” conformation (PfAgo in its free state (mint green) with the “unplugged” glutamate finger, PDB: 1U04; cleavage-incompatible ternary TtAgo complex with “unplugged” glutamate finger (PDB: 3F73, corn blue); cleavage-compatible ternary TtAgo complex with “plugged” glutamate finger (PDB: 3DLH, orange); ternary hAgo2 complex with “plugged” glutamate finger (PDB: 4W5T, grey). In the “plugged” conformation, an invariant glutamate sidechain is inserted to complete the tetrad in the catalytic pocket (the broken arrow indicates the relocation of E512).

The Argonaute (Ago) protein family was initially discovered in eukaryotes [1,2], but orthologs were found in many archaeal and bacterial organisms [3,4,5]. In eukaryotic organisms, Argonaute represents the principal component of the RNA silencing machinery. Despite the advancements in the understanding of Argonaute function in the eukaryotic field, the biological role of prokaryotic Argonaute proteins (pAgo) remained unknown for a long time. Argonaute proteins are encoded in ~32% and 9% of the sequenced archaeal and bacterial genomes, respectively [6]. PAgos were found to cluster in two groups distinguished by the presence or absence of the PAZ domain [5]. A lack of the PAZ domain often coincides with an apparent inactivation of the nuclease activity [5,6]. Interestingly, pAgos are often found in operons with a diverse range of endonucleolytic DNases (nucleases of the restriction endonuclease fold, a distinctive Sirtuin family domain or TIR domain proteins) and/or helicases (e.g., of the DinG-class) [3,5,7], leading to the hypothesis that the co-action of pAgo and an endo-DNase might act as a plasmid/phage restriction system. However, the subset of pAgos that exhibit a high sequence similarity to their eukaryotic counterpart does not seem to show conserved operonic associations with any other genes. Ago is composed of the N-terminal, PAZ (Piwi-Argonaute-Zwille), middle (MID) and PIWI (P-element-induced wimpy testis) domains interconnected by two structured linker regions (Figure 1 and Figure 2). This review mainly discusses the “long” pAgo variants, which share a comparable domain organization as determined for the eukaryotic Agos. In contrast, short pAgos variants contain the MID and PIWI domain only [5]. Recently, two studies have shed light on the biological role of bacterial Agos [8,9]. Together with our findings on the substrate specificity of an archaeal Ago variant [10], these data point to a paradigm shift in the field, as the spectrum of Argonaute silencing activities now also includes DNA- or RNA-guided DNA interference in prokaryotic organisms.

## 2. Structural Organization of Argonaute

The structures of archaeal [11,12] and bacterial [13,14,15] Agos provided valuable insights into the structure-function relationship of the protein before structures of eukaryotic Ago became available [16,17,18]. The structure of Argonaute from the archaeal organism *Pyrococcus furiosus* (PfAgo) was the first reported full-length structure [12], quickly followed by the structures of bacterial and, finally, eukaryotic Argonaute variants. Interestingly, the Argonaute structures from all three domains of life show a high degree of similarity (Figure 1). The domains of the protein are arranged in a bilobal fashion with the PAZ and N-terminal domain forming one and PIWI and MID domains the other lobe. The 5'-end of the guide strand is buried in a deep pocket at the interface between the MID and PIWI domains in a highly conserved region where side chains of four invariant residues contact the obligatory 5'-end phosphate group of the first nucleotide. These four residues are highly conserved among archaeal and eukaryotic Agos. In the *A. fulgidus* PIWI (AfPIWI) protein, these residues are Y123, K127, Q137 and K163 [19], whereas in human Ago2 (hAgo2), the terminal phosphate is stabilized by Y529, K533, Q545 and K570 [17,20,21]. The *T. thermophilus* Ago (TtAgo), in contrast, uses different residues, which are R418, K422, S432, Q433 and K457 [15]. The insertion of the 5' nucleotide into the binding pocket of TtAgo leads to a distortion of the RNA/DNA duplex, which results in an unwinding of the first base pair of the loaded nucleic acid duplex [15,19,22]. Interestingly, there a no extensive base-specific contacts between amino acid side-chain residues and the bases. However, structures of TtAgo with a 21 mer guide DNA with complementary 12-mer target RNA or target DNA show that the terminal 5'-end nucleotide of the let-7 guide (a thymine) undergoes hydrogen-bonding contacts with the backbone amide carbonyl of residue M413 and the side chain of N436 [14,23]. For the eukaryotic Argonaute, a nucleotide specificity loop contributes to the recognition of the first nucleotide (Figure 2a). The specificity loop can be found in archaeal and bacterial Agos, but not all residues in the loop are conserved [21]. The loop is pulled away from the nucleotide of the base in the AfPIWI structure [24], while AMP and UMP interact with the backbone atoms of the hAgo2 specificity loop (G524 and T526). In contrast, GMP and CMP are repulsed by the loop [21]. The structures of AfPIWI and TtAgo show that a divalent metal ion is coordinated with the C-terminus of Argonaute. This metal ion is involved in 5'-end binding [15,22], while eukaryotic Argonautes do not make use of a metal ion and neutralize the charge by a lysine side chain [16]. The electron density of the guide strand can be detected easily for nucleotides 2 to 7 or 8 (the “seed” region [13,18,20,25,26]). Here, amino acids located in the MID, PIWI domain (R792, K709, Y804, S798, R761) and L1 linker (A221) of hAgo2 contact the guide strand via salt linkages to the phosphate backbone and hydrogen bonding [20]. The nucleotides in the seed region are continuously base-stacked and solvent-exposed, while the nucleotides beyond nucleotide 7 are threaded into Ago [17,18]. None of the contacts establish any sequence specificity congruent with Argonaute’s ability to bind a multitude of different guide sequences.

Beyond the seed region, the structure of the guide strand appears to be largely disordered, with the exception of the guide’s 3'-end. The structures of the TtAgo and hAgo2 binary complex showed that both proteins introduce a pronounced kink in the guide strand. Kinking after nucleotide 6 at the end of the seed region in hAgo2 is caused by I365 [14,16,17,27]. In TtAgo, a similar kink is positioned at nucleotide 10 of the guide [15]. The last two nucleotides of the guide strand are contacted by conserved aromatic and basic residues anchoring the 3'-end in the PAZ domain [15,16,17,19,20,28,29] (Figure 2b). There is no structural information of the archaeal binary complex available, but single-molecule measurements in solution using the archaeal Argonaute protein from *Methanocaldococcus jannaschii* (MjAgo) provide evidence for a 3'-end anchoring of the guide strand in the archaeal domain [10]. Kinetic studies revealed that the binding of hAgo2 to a guide strand follows a consecutive pathway. The association occurs in a three-phase process. The first phase, which is only limited by diffusion, represents a collision between hAgo2 and a guide strand. The following phase corresponds to the subsequent anchoring of the guide’s 5'-end in the MID domain, which is a pre-requisite for the third phase, representing the docking of the guide’s 3'-end in the PAZ-domain [30]. Therefore, Argonaute provides a scaffold for the integration of a guide strand, making it plausible that guide recognition follows a highly conserved mechanism.

While the guide strand is firmly anchored in Ago, integration of the target strand is mainly mediated via Watson–Crick base pairing with the guide strand. The bases of the guide in the seed region face outwards, ready to base pair with a target. Thus, the target strand associates with the pre-arranged guide strand, forming an A-form helix in the seed-region. This nucleation process does not require an extensive interaction network between the protein and the target strand [13,14]. Instead, specificity is mediated by the shape complementarity between Argonaute and the minor groove of the duplex, which allows for hydrophobic and van der Waals interactions of the linker 2 domain with the guide-target duplex in the seed region [18]. The guide-target duplex extends from the seed towards the 3'-end binding site. The duplex remains in a helical form before further base pairing is disrupted in the N-PAZ channel, where the helical form is blocked by the N-terminal domain. Bacterial structures showed that the strand separation occurs at nucleotide 16 of the guide strand [14]. The unstacked bases of the guide face into the interior of the complex, preventing an association of the target strand in the 3' half of the duplex. The catalytic center resides in the PIWI domain, which adopts a typical RNase H fold. The catalytic activity is mediated by a catalytic tetrad composed of a highly conserved DEDX (X being an aspartate or histidine). In addition, parts of the N-terminal domain influence the cleavage activity of human Argonautes [31,32,33,34]. Here, unstructured loops seem to arrange the target strand in a cleavage-compatible geometry. 

While the overall structural organization of Ago across the domains of life is highly similar, there are also significant differences noticeable. The individual Ago domains align well, but the relative position of the lobes differs significantly. PfAgo adopts a more compacted form than hAgo2 and TtAgo, with the PAZ and MID module moved towards each other (Figure 1). However, the unstructured loops found in the eukaryotic N-terminal domain necessary for cleavage activity are significantly shortened or non-existent in the archaeal counterparts. Eukaryotic Ago proteins show a number of additional loops and unstructured elements [16,17,35]. Most of these elements are surface-exposed, representing contact sites for eukaryotic-specific interaction partners [36]. A significant difference between eukaryotic and archaeal-bacterial Argonautes can be found in the surface charge distribution. The surface of the duplex binding channel, as well as the binding pocket in the MID domain of eukaryotic Ago is strongly positively charged (Figure 1). In contrast, the binding channel of TtAgo is less positively charged (Figure 1), and the binding pocket is more hydrophobic [17]. This is even more pronounced in PfAgo. An extended negatively-charged surface can be found in the N-PAZ tunnel of PfAgo, which might hint at an alternative pathway for the guide or target strand after the release of the guide strand from the PAZ domain.

## 3. Conformational Flexibility Facilitates Argonaute Function

Argonaute does not merely represent a static scaffold structure that assists nucleic acid strand association and dissociation, but the protein itself undergoes dynamic changes throughout its activity cycle. The crystal structures of the binary and ternary complex, pre-steady state kinetics and single-molecule experiments revealed the presence of flexible domains, loops and helices that promote the positioning of the target strand in a cleavage-compatible configuration. The most pronounced conformational change occurs on the progression from the binary to the ternary complex, as captured for TtAgo (Figure 2b). TtAgo structures in complex with a 21-mer DNA guide and DNA or RNA target strands showed that upon loading of a sufficiently long target strand, the 3'-end of the guide is released from the PAZ domain [14,23]. Single-molecule studies using TtAgo, a DNA guide and an RNA target strand even suggested that the release of the 3'-end might be a dynamic event [37]. The rearrangement of the 3' half of the guide is accompanied by a significant movement of the PAZ domain. Meanwhile, the 5'-end of the guide strand remains firmly anchored in the MID binding pocket [14]. The release from the PAZ domain is a direct consequence of the helical structure of the DNA that prevents the 3'-end from reaching the PAZ binding pocket. Hence, PAZ release is correlated with the length of the duplex. TtAgo structures reveal the influence of the target substrate on the duplex length. A 15-mer target RNA leads to a DNA guide-RNA target duplex with a length of 14 bp, which is accompanied by the release of the guide 3'-end from the PAZ domain [14]. In contrast with a 15-mer target DNA, the DNA guide-DNA target duplex spans only 13 bp, with the guide 3'-end still being anchored in the PAZ domain. A 16-mer DNA target leads to the formation of 15 bp between guide and target DNA and induces the release of the guide 3'-end from the PAZ domain. Therefore, with TtAgo, the rearrangement of a cleavage-incompatible to a cleavage-compatible conformation differs for RNA and DNA target substrates [23]. However, incorporation of a short target strand already induces a conformational change in the protein, leading to the opening of the PAZ domain [14]. A more pronounced rotation of the PAZ domain in a situation where the nucleic acid duplex is further extended results in a widening of the nucleic acid binding channel to accommodate the target strand [14,23]. A comparable ternary structure of eukaryotic Argonaute variants, including a full-length target RNA, could not be solved yet. However, analogous to the bacterial structures, hAgo2 loaded with an RNA guide only or a short RNA duplex (11-nt target) shows the 3'-end still anchored in the PAZ domain [18]. Nevertheless, kinetic experiments suggest that extended pairing of the guide and target most likely also leads to 3'-end release [30]. Even though there are no structures of the binary or ternary complex available for the archaeal domain, recent single-molecule fluorescence resonance energy transfer (FRET) studies on the archaeal Argonaute protein from *Methanocaldococcus jannaschii* in solution support the common theme of PAZ release upon the formation of the ternary complex using a DNA duplex (21-mer guide, 20-mer target) [10]. This study also showed that the release of the guide 3'-end in the archaeal enzyme does not require cleavage of the target strand. Taken together, these data support the two-state model of Argonaute action [38]; a mechanism conserved in Argonaute variants from all domains of life.

Integration of the target strand in TtAgo leads furthermore to the straightening of the guide DNA to fully adopt an A-form with consecutive base-stacking. As a result, bases 10 and 11 of the guide strand bound by TtAgo stack on top, allowing the correct orientation of the scissile phosphate of the target strand relative to the catalytic residues [14]. The hAgo2 structures also show that the kink between nucleotides 6 and 7 of the guide is relieved due to a movement of helix α7, which is necessary to avoid steric clashes with the target strand (Figure 2). Helix α7 and the PAZ domain move as discrete rigid bodies relative to the MID, PIWI and N-terminal domain upon target loading [18]. Interestingly, helix α7 is conserved in archaeal, but not bacterial Ago [17]. A significant conformational transition occurs close to the active center of the enzyme with direct consequences on the active site configuration. In TtAgo, PIWI loop 2 undergoes a conformational switch, thereby inserting a “glutamate finger” into the active site, completing the catalytic tetrad (Figure 2c). Strikingly, all TtAgo structures with the 3'-end located in the PAZ domain show the “unplugged” conformation, while all PAZ-released structures show the “plugged” conformation of the glutamate finger. Hence, the PAZ release seems to be coupled to the activation of the slicing activity of the enzyme. The archaeal structure of unliganded Argonaute exhibits an unplugged conformation. The unplugged to plugged transition is not found in eukaryotic Agos: all structures available show the glutamate finger in the plugged conformation, irrespective of the PAZ release [16,17,18,35].

## 4. Molecular Mechanism of the Silencing Process

### 4.1. RNA Interference Mediated by Human Argonaute 2

In eukaryotes, Argonaute constitutes the principal component of the eukaryotic RNA interference (RNAi) pathway, a mechanism fundamental to posttranscriptional regulation of gene expression [39]. Generally, in the RNAi system, Argonaute 2 is loaded with short double-stranded RNAs within the RLC (RISC-loading complex) [40,41]. One strand of the RNA duplex (the guide strand) is retained in the Argonaute protein, which is part of a multiprotein complex, called RISC (RNA-induced silencing complex). The non-guide strand (passenger strand) is cleaved by Argonaute and eventually ejected, allowing the guide strand to find its cognate mRNA target [25,31,42]. The fully complementary target RNA of a siRNA-guided RISC is cleaved by hAgo2 (slicing) and released, and RISC can engage in another round of slicing [43,44]. In contrast, miRNAs typically guide hAgo2 to partially complementary targets in the 3' untranslated regions (UTR) of mRNAs, which leads either to mRNA degradation by hAgo2 or to translational inhibition [45,46,47]. Extensive research over the last two decades has shown that the function of Argonaute exceeds its role in RNA silencing (for recent reviews, see [36,48,49]) and revealed its role in chromatin dynamics [50], transcriptional regulation [51,52], alternative splicing [53,54,55] and double-strand break repair [56]. Eukaryotic organisms frequently encode more than one Argonaute gene. The nematode *C. elegans* encodes an impressive Argonaute family of 27 members [57,58]. However, not all Argonautes proteins are catalytically active variants. Among the four human Agos, only hAgo2 acts as a nuclease [59,60].

### 4.2. Prokaryotic Argonaute Acts in DNA-Silencing Pathways

**Figure 3:**
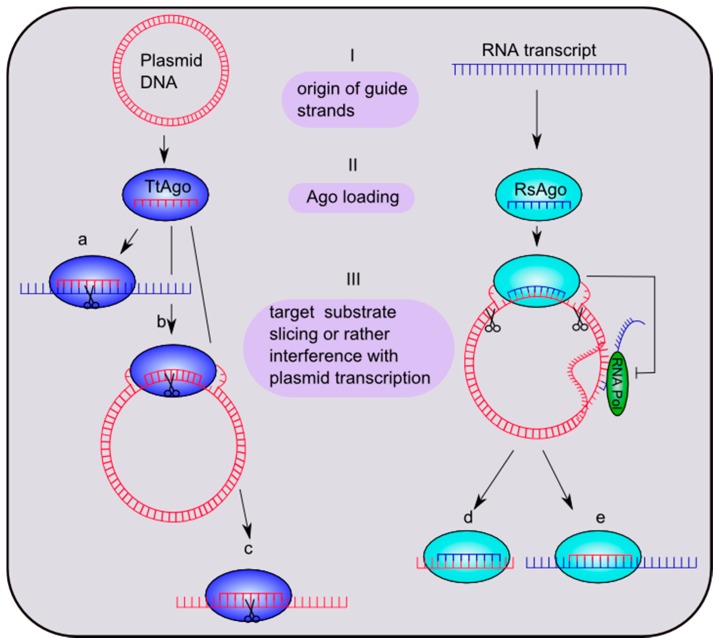
Putative mechanisms of bacterial Ago-mediated silencing pathways. (I) Guide sequences of *Thermus thermophilus* Ago (TtAgo) and *Rhodobacter sphaeroides* Ago (RsAgo) are derived from plasmid DNA or RNA transcripts, respectively. (II) TtAgo is loaded with a 13–25-nt guide DNA and RsAgo with a 15–19-nt guide RNA. (III) The target substrates are (**a**) ssRNA, (**b**) negatively-supercoiled plasmid DNA and (**c**) ssDNAs in the case of TtAgo. RsAgo binds plasmid DNA, which will be cleaved, yielding 22–24-nt DNA fragments. Binding of the guide-Ago complex to plasmid DNA furthermore possibly leads to an inhibition of plasmid transcription. The short fragments either stay bound to Argonaute (**d**) or interact with other RsAgo molecules to constitute DNA-RsAgo complexes and regulate plasmid transcription (**e**). Figure in part modified from Olovnikov *et al.* [8].

Despite the mechanistic and structural similarities between the archaeal, prokaryotic and eukaryotic Argonaute proteins, a major difference in the fundamental mechanism of silencing was revealed recently. Bacterial Argonautes use either DNA or RNA guide strands to silence complementary DNA strands (Figure 3). In native cells Argonaute from the alphaproteobacterium *Rhodobacter sphaeroides* (RsAgo) is associated with small RNAs and DNAs [8]. Interestingly, RsAgo belongs to the Ago class with an inactivated catalytic tetrad. However, RsAgo is encoded in an operon with a predicted DNA nuclease. The small RNAs are derived from mRNA precursors that can be mapped to the majority of cellular transcripts and most likely are generated from mRNA degradation products. DNAs associated with RsAgo are largely complementary to the bound RNAs. A current model for the generation of RNA-interacting DNAs (riDNA) proposes that the small RNA directs RsAgo to the complementary DNA target followed by the nucleolytic cleavage of the DNA by a yet unidentified nuclease. Alternatively, RsAgo-RNA complexes loaded onto a complementary stretch of DNA inhibit RNA polymerase loading or block RNA polymerase elongation, leading to transcriptional repression of the target DNA. The enrichment of riDNA for foreign sequences, like plasmid DNA and transposons, suggests that the RsAgo-mediated DNA silencing mechanism is in place to destroy foreign genetic elements. However, the molecular mechanisms that allow the discrimination between self and foreign DNA are not known. Another example of bacterial Ago-mediated DNA silencing was described for TtAgo. Expression of TtAgo in *E. coli* and subsequent characterization of TtAgo-bound nucleic acids revealed that TtAgo associates primarily with DNA sequences (small interfering DNAs) preferentially derived from its own expression plasmid [9]. The underlying mechanism for DNA guide processing from foreign DNA (e.g., plasmids) has not been deciphered yet. However, loading of TtAgo with guide DNA and subsequent cleavage of target DNA is only observed if the catalytic center of the enzyme is intact, indicating that the nuclease activity of TtAgo is required for guide processing. Even though the experimental characterization of TtAgo *in vivo* was mainly carried out using plasmid DNA, it is feasible that the DNA-guided DNA silencing mechanism targets replication intermediates from invading genetic elements and DNA taken up by the natural competence system present in *Thermus thermophilus*. The functional role of archaeal Argonaute still remains elusive. However, *in vitro* studies showed that MjAgo exclusively cleaves DNA targets when using a DNA guide [10], suggesting that this archaeal Argonaute variant, like the bacterial counterparts, is involved in DNA silencing processes.

### 4.2. Diversity in Recognition and Selection of Guide and Target Strands

Most eukaryotic and prokaryotic Agos are able to associate with RNA, as well as with DNA substrates [10,14,61], but act in a very selective way *in vivo*. Thus, what are the determinants that guide the selection of small nucleic acid interaction partners for Ago proteins? Signatures like length, phosphorylation status and the identity of the first nucleotide, play an important role in selection and sorting (see Table 1 for an overview). HAgo2 preferentially associates with small RNAs 20–25 nucleotides in length. Even though hAgo2 tolerates a variety of guide lengths, lengths of 21 nt (siRNA) [43,62] or 22 nt (miRNA) [44] are most abundantly associated with hAgo2. Furthermore, dsRNAs associated with hAgo2 are distinguished by 2-nt overhangs, a 5'-end phosphate group and a 3'-end hydroxyl group [63]. These signatures are a result of the biogenesis pathway [64]. Central to si/miRNA biogenesis are Drosha and Dicer, two nucleases that process dsRNA substrates into short dsRNA fragments, typically of 21–25 nt in length [65]. Drosha is involved in the maturation of the 5'-end, creating the characteristic phosphate group at the 5'-end. Structural information on Dicer came from the Doudna lab, which was able to solve the structure of the enzyme derived from the unicellular eukaryote *Giardia intestinalis* [66]. The structure revealed why Dicer cleavage products (i) have a certain length and (ii) possess a 2-nt overhang at the 3'-end. The RNase III domains form an intramolecular dimer, and the active centers are located opposite, but slightly shifted to each other, separated by 17.5 Å, which matches the width of a major groove in dsRNA, making it plausible that Dicer generates dsRNAs with a 2-nt overhang. Recent structural and functional studies revealed that Dicer not only anchors the 3'-end, but simultaneously the 5'-end of the RNA substrate in a pocket in the platform domain of Dicer [67,68]. 5'-end docking is especially important to generate uniform 22 nt-long products (5' counting rule).

**Table 1:** Substrate preferences of prokaryotic Argonautes proteins *in vitro* and *in vivo*. n.d. = not determined; inactive = no catalytic tetrad present; ( ) = significantly reduced affinity; ^#^ = determined *in vitro*; * = determined *in vivo*; { } unpublished.

Argonaute Variant	Guide Strand Bound	Preference/Enrichment For 1st Guide Nucleotide	Target Strands Bound	Target Strands Cleaved	Ref.
**Archaea**					
*M. jannaschii* Ago	DNA ^#^, (RNA) ^#^	{G} ^#^	DNA ^#^, RNA ^#^	DNA ^#^	[10]
*A. fulgidus* Ago	DNA ^#^, (RNA) ^#^	n.d.	DNA ^#^, RNA ^#^	n.d.	[19]
**Bacteria**					
*A. aeolicus* Ago	DNA *, (RNA) *	n.d.	RNA ^#^, DNA ^#^	n.d.	[69]
*R. sphaeroides* Ago	RNA *, DNA *	U *	RNA *, plasmid DNA^#^	inactive	[8]
*T. thermophilus* Ago	DNA *^,#^, (RNA) ^#^	C *	DNA *^,#^, RNA ^#^, plasmid DNA *^,#^	DNA ^#^, RNA ^#^, plasmid DNA *^,#^	[9,14]

Stable anchoring of the 5'-end requires a 5' phosphate, which is the result of pre-miRNA processing by Drosha. The isolated MID domain of hAgo2 binds AMP and UMP with up to 30-fold higher affinity as compared to CMP and GMP [21]. A preference for a uridine as the first nucleotide in eukaryotic Ago-associated RNA was observed in several studies indicating that the first nucleotide serves as a determinant for guide selection [16,57,64,70,71]. In plants, small RNA sorting in the different Agos is predominantly determined by the identity of the 5' nucleotide [59,72]. Structural studies have provided the structural basis for the 5' nucleotide specificity [21,73,74] that is conferred by the nucleotide specificity loop (Figure 2a). The 5' terminal nucleotide packs against a tyrosine residue (Y529), which contributes to the non-specific recognition of the base. In addition, specific interactions with a threonine (T526) and glycine residue in the specificity loop are only possible if the base is an UMP or AMP. The specificity loop is missing in the bacterial Ago variants, and only residue N436 undergoes a specific interaction with the first nucleotide (TtAgo in complex with the let-7 DNA guide and the DNA or RNA target) [14,23]. However, specific interactions between the protein backbone and the nucleotide cannot be established in AfPIWI and PfAgo, as the specificity loop is arranged too far from the 5'-nucleotide [22,24] (Figure 2a). It remains to be determined whether there are additional factors that determine the nature of the 5'-nucleotide where a 5'-nucleotide preference occurs, albeit the nucleotide bias appears not to follow a common pattern. For example, it would be conceivable that a pre-processing enzyme generates DNA guides with a defined 5'-nucleotide.

Information about the nucleic acid interactions partners associated with prokaryotic Ago *in vivo* are only available for bacterial Ago variants [8,9], as the growth and manipulation of archaeal organisms that encode potentially active Argonautes are notoriously difficult. However, *in vitro* studies on the recombinant archaeal MjAgo showed that the protein binds short nucleic acids with the characteristic siRNA/miRNA signatures, e.g., a 5'-end phosphate on the guide strand and two nucleotide overhangs, irrespective of the nucleic acid chemistry. However, MjAgo preferentially binds and exclusively cleaves DNA/DNA hybrids [10]. Furthermore, MjAgo exhibits a preference for a deoxyguanosine as the first nucleotide in the guide strand (unpublished data). A similar preference for DNA substrates was found for the isolated PIWI domain of *A. fulgidus* [19]. These data hint to the possibility that archaeal Agos, like their bacterial counterparts, act in a DNA-guided DNA silencing mechanism.

DNA-guided DNA silencing has been described for the bacterial Ago from *Thermus thermophilus* in *in vitro* and *in vivo* experiments [9]. TtAgo loaded with DNA cleaves DNA and RNA substrates *in vitro* [14]. An overhang of the target at the 3'-end of the target is not required for efficient cleavage [14]. When expressed in *E. coli*, TtAgo associates with DNA guides 13–25 nt in length with a strong bias towards 15-nt guides. Furthermore, the guide DNA strands appear to carry the typical 5'-end phosphate. Interestingly, the majority of guides have a deoxycytidine and a deoxyadenosine at the first and second nucleotide, respectively. These guides enable TtAgo to cleave double-stranded DNA, supporting the idea that TtAgo follows a DNA-guided DNA silencing mechanism [9] (Figure 3). However, bacterial Argonautes do not follow a common selection pattern. *A. aeolicus* Ago shows the highest affinity for DNA as a guide and for a DNA/RNA as a hybrid [69]. In contrast, RsAgo favors 5'-end phosphorylated 18-nt RNA guide strands and 24-nt DNA target strands. The first and second nucleotide of the guide shows a strong enrichment for a uridine. The target strand is fully complementary to the guide strand, but shows an unusual 3-nt overhang on both sides. It is difficult to rationalize the selection patterns of prokaryotic Argonautes, as no homologues of Drosha or Dicer, which act further upstream of Argonaute and could distinguish between RNA or DNA precursors, are encoded. Heterologous expression of RsAgo in *E. coli* leads to specific loading of 5'U-RNAs with the correct length from a pool of available sequences and, hence, might indicate that no pre-processing machinery is needed for RNA-guided DNA silencing by RsAgo [8].

## 5. An Archaeal Perspective

While the biological function of Argonaute proteins is not conserved, many structural features and conformational changes that support the activity cycle of Argonaute are well-preserved across domains. Among them is the ability to bind short DNA or RNA guide strands with a preference for a terminal phosphate at the 5'-end of the guide that ensures stable incorporation of the guide into the MID domain. In addition to structural features, conformational dynamics are also conserved, suggesting that they play an important role for the function of the protein. Structural and biophysical studies demonstrated that the PAZ domain is a mobile element in all Argonaute variants [10,13,30,37]. However, some of the structural features are exclusively shared between archaeal and eukaryotic Argonaute variants. Helix α7 is not conserved in the bacterial domain, and the interaction network that allows the recognition of the first nucleotide at the 5'-end of the guide is well preserved between archaeal and eukaryotic Argonautes. The recently discovered DNA silencing function of bacterial Agos raises the question of whether comparable mechanisms are in place in archaeal organisms. However, while archaeal proteins served as an excellent model system to elucidate the structure of the Argonaute family, catalytically active forms were rarely characterized *in vivo* or *in vitro*. In addition, the structure of an archaeal Argonaute in complex with nucleic acid substrates is still lacking. Experiments employing the Argonaute protein from the hyperthermophilic organism *Methanocaldococcus jannaschii* suggest, however, that some archaeal Argonautes support a DNA-guided DNA silencing mechanism. From an archaeal perspective, there is still much to explore about the structure and function of Argonautes, and further studies are needed to discover the full picture of the Argonaute family.
